# Expression level of *CEBPA* gene in acute lymphoblastic leukemia individuals

**DOI:** 10.1038/s41598-019-52104-w

**Published:** 2019-10-30

**Authors:** Dagmara Szmajda, Adrian Krygier, Krzysztof Jamroziak, Marta Żebrowska-Nawrocka, Ewa Balcerczak

**Affiliations:** 1 Laboratory of Molecular Diagnostics and Pharmacogenomics, Department of Pharmaceutical Biochemistry and Molecular Diagnostics, Medical University of Lodz, Lodz, Poland; 2 Institute of Hematology and Transfusion Medicine, Warsaw, Poland

**Keywords:** Acute lymphocytic leukaemia, Molecular medicine, Transcriptional regulatory elements, Gene expression, Acute lymphocytic leukaemia

## Abstract

Currently, acute lymphoblastic leukemia (ALL) has an overall survival of nearly 80% when it occurs in children, however cure rates among adults are far reduced. Leukemogenesis can be driven up by a slight change in the expression or function of certain transcription factors. CCAAT Enhancer Binding Protein Alpha (*CEBPA*) is a transcription factor with role in cell cycle regulation, granulocytic differentiation and more. Some studies suggest its oncogenic function. The potential role of *CEBPA* as an oncogene in ALL development has not been completely elucidated so far. Therefore, the aim of the present study was to evaluate mRNA level of *CEBPA* gene in 60 adult patients diagnosed with ALL. Quantitative analysis was performed by qPCR reaction. Analysis revealed that men tended to have higher and more variable *CEBPA* expression levels (*P* = *0.032*). No associations for other parameters (ALL subtype, age, leukocytosis, blast percentage, Philadelphia chromosome presence, CD10 marker presence) were found. When comparing the results of *CEBPA* expression with patients suffering from acute myeloid leukemia, ALL cases showed statistically significant lower levels of *CEBPA* (*P* < *0.0000*). It may seem that *CEBPA* expression level itself has potentially no effect on arising and progression of acute lymphoblastic leukemia, although it is a matter that needs further investigation.

## Introduction

Acute lymphoblastic leukemia (ALL) is a malignant disease of lymphoid progenitor cells affecting both children and adults^1^. At present, ALL is successfully curable in almost 80% of cases if it comes to children, however, it shows poor prognosis when arising in adults^2,3^. Despite the high cure rates, the complexity, expenditure and toxic side-effects of contemporary multidrug treatments must be mentioned^2^. Thus, it is advisable to continue analyzing genetic abnormalities of leukemic cells and to transform them further into enhanced diagnostic methods, targeted novel therapeutics and treatment strategies^1,4^. Furthermore, specific predisposing factors leading to acute lymphoblastic leukemia development are not fully known yet, however, leukemogenesis can be driven up by a slight change in the expression or function of certain transcription factors^4^.

CCAAT Enhancer Binding Protein Alpha (*CEBPA*) is an intronless gene located on chromosome 19q13.1^5,6^. It encodes protein belonging to transcription factors family containing a basic leucine zipper (bZIP) motif^6,7^. CEBPA function includes the following modulation of the genes expression involved in the cell cycle regulation and body weight homeostasis^8–10^, it is also crucial in granulocytic differentiation^10,11^ and probably acts as a tumor suppressor in hematologic and non-hematologic malignancies^10^. On the other hand, some results indicate that *CEBPA* may act as an oncogene in lymphoid malignancies, in contrast to its role as a tumor suppressor in myeloid leukemia^12^. The potential role of *CEBPA* as an oncogene in ALL development has not been completely elucidated so far. Therefore, the aim of the present study was to evaluate mRNA level of *CEBPA* gene in patients diagnosed with ALL.

## Results

### *CEBPA* expression level according to patients’ gender and age

The mRNA expression level of *CEBPA* gene was successfully quantified in 60 patients. *CEBPA* transcript level varied among selected cases, it ranged from 3.70 to 14.64 with a mean value 7.97 overall, from 4.04 to 14.64 with a mean value 8.41 for women and from 3.70 to 10.69 with a mean value 7.21 for men. Detailed clinic-pathological parameters of patients are presented in Table 1.

**Table 1:** Clinic-pathological characteristics of the investigated cohort.

Parameters	Number of cases (n = 60)
Gender	
Men	22
Women	38
Age (years)	
Range (mean)	35–81 (58)
Leukemia subtype	
B-ALL	47
T-ALL	13
Leukocytosis	
Range (mean)	17000–31000 (23732)
Blast percentage	
Range (mean)	67–99 (83)
Philadelphia Chromosome	
Positive	4
Negative	56
CD10 marker	
Positive	6
Negative	41

**Figure 1:**
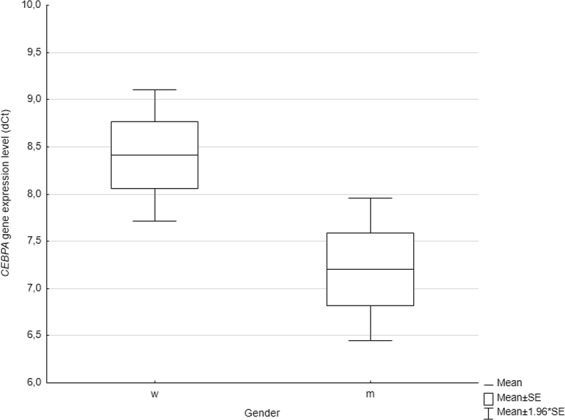
Association between the *CEBPA* expression level and gender. Men presented lower dCt values and thus tended to have higher expression level (P < 0.05).

The dependence between patients’ gender and the *CEBPA* expression level was then sought, 38 women and 22 men were included in the study. The analysis revealed that there was a statistically significant difference in selected gene transcript level (*P* = *0.032*) depending on gender. Males tended to have higher and more variable *CEBPA* expression levels (Fig. 1).

Next, the *CEBPA* expression level according to age of the investigated cohort was rated, the group ranged from 35 to 81 years of age, with a mean value of 58 years of age. No statistically significant correlation was obtained (*P* = *0.684*).

### *CEBPA* expression level according to leukemia subtype and hematological parameters

Patients were then divided into two groups, according to leukemia subtypes, to B-ALL (47 cases) and T-ALL (13 cases). Transcript level of *CEBPA* ranged from 3.70 to 14.64 with a mean value 7.92 for B-ALL subtype and from 5.18 to 12.47 with a mean value 8.13 for T-ALL. No meaningful difference was found between these two subgroups of patients (*P* = *0.763)*.

After that, the dependencies between leukocytosis, blast percentage in the bone marrow and *CEBPA* transcript level were evaluated. Statistical analysis exhibited no significant differences between the selected parameters and *CEBPA* level (*P* = *0.693, P* = *0.620*, accordingly).

Also, the comparison between Philadelphia chromosome presence and the selected gene expression level was done, Ph chromosome was found in 4 cases, however, no statistically significant differences were found between Ph bearers and non-bearers (*P* = *0.119*).

Amongst the B-ALL patients the presence of CD10 marker was additionally evaluated, out of 47 cases 6 were CD10-negative. Analysis revealed no significant differences in mRNA *CEBPA* level between CD10-positive and CD10-negative trials (*P* = *0.169*).

### Comparison of *CEBPA* expression level between ALL and AML patients

**Figure 2:**
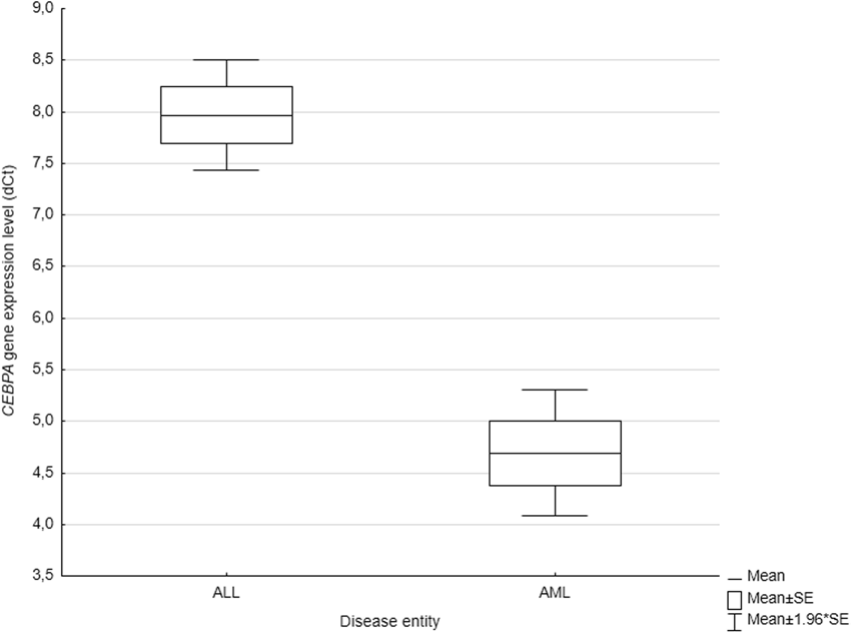
Differences in *CEBPA* gene expression level between AML and ALL. Acute lymphoblastic leukemia cases presented higher dCt values and thus lower expression level when compared to acute myeloid leukemia group (P < 0.0000).

Additionally, the difference in *CEBPA* expression level was evaluated between investigated ALL cohort (n = 60) and 45 adult AML cases, geographically and ethnically matched. Obtained results indicate a significant dissimilarity in *CEBPA* transcript level between the two acute leukemias (*P* < *0.0000*), in particular investigated ALL group presented lower *CEBPA* expression (Fig. 2).

## Discussion

Our knowledge about genomics of ALL still remains incomplete. This investigation was carried out in order to answer the question whether *CEBPA* gene expression level has any association with clinic-pathological features and influences the process of leukemia development/progression in individuals with ALL. Research conducted by Grossmann *et al*. indicated that expression of *CEBPA* is lower in AML (acute myeloid leukemia) patients with mutated *RUNX1* (Runt Related Transcription Factor 1) gene^13^. On the other hand, Gholami *et al*. performed the evaluation of *CEBPA* expression and showed its significant upregulation in AML patients compared to healthy controls^14^. Similar observation has been made in our analysis, where AML cases showed higher *CEBPA* levels when compared to ALL patients (P < 0.0000). Interestingly, Gholami *et al*. also found that significantly higher levels of *CEBPA* were observed in adult male AML patients^14^. This finding is consistent to our study, where men presented up-regulated and more variable *CEBPA* mRNA levels. There are difficulties in comparing the obtained results since no similar studies on ALL cases have been carried out so far. Chapiro *et al*. found that *CEBPA* is overexpressed in patients with (14;19) (q32;q13) chromosomal translocation suffering from precursor B acute lymphoblastic leukemia^12^. This translocation was absent in the studied cohort, therefore comparison was not feasible. In addition, the study group included both T-ALL and B-ALL cases. Perotti *et al*. found that in Ph-positive patients with CML (chronic myeloid leukemia) blast crisis CEBPA protein expression is down-regulated, while it maintains normal mRNA levels^15^. In our study we did not detect a statistically significant difference in *CEBPA* mRNA levels among ALL subjects with and without *BCR-AB*L fusion gene presence, however the Ph bearers group is small and no final conclusion can be drawn. Further trials in this field are highly desirable.

It may seem that *CEBPA* expression level itself has potentially no effect on arising and progression of acute lymphoblastic leukemia, although it seems to alter the leukemogenic process among women and men, since men tended to have higher mRNA levels. Overall ALL cases show lower *CEBPA* transcript levels, this seems to sustain the hypothesis that both a reduction and increase in gene dosage may be involved in the pathogenesis of various leukemia types. Patients with *BCR-ABL* fusion gene presence are an interesting aspect requiring further investigation. We are aware of several limitations of our study. The primary limitation of obtained results is the extraction of RNA from whole, but not ficolled or lymphopreped blood. Although the blast percentage among investigated group was high, there could be contamination of other cell types, which may have affected observed expression levels. It should be stated that the presented outcomes are limited to the Polish population with restricted sample size. To confirm the results, further studies are required on larger groups of ALL patients from diverse populations.

## Material and Methods

For the investigation, patients diagnosed with ALL have been recruited. 60 blood samples collected from patients comprised the research material. The present study was approved by The Ethics Committee of the Medical University of Lodz (Number RNN/88/16/KE) and was in accordance with the principles of the Declaration of Helsinki. Written informed consent was obtained from the patients prior to their participation in the research.

Firstly, RNA was isolated from peripheral blood of patients, according to the manufacturer’s protocol, using Total RNA Mini kit (A&A Biotechnology, Gdynia, Poland), which is based on modified phenol-chloroform extraction with fenozol. Then the reverse transcription reaction was performed in order to obtain cDNA, using a High Capacity cDNA Reverse Transcription kit (Applied Biosystems; Thermo Fisher Scientific, Inc., Waltham, MA, USA), according to the manufacturer’s protocol. The reaction parameters were as follows: 25 °C for 10 min, 37 °C for 120 min and 85 °C for 5 min. The amount of RNA was equated and set to 0.2 μg per sample. Quantitative analysis was performed by qPCR reaction, using Rotor-Gene 6000 apparatus (Corbett Life Science; Qiagen GmbH, Hilden, Germany). The following primes were used in the study: for the investigated gene – *CEBPA* forward 5′-AGCCTTGTTTGTACTGTATG-3′, reverse 5′-AAAATGGTGGTTTAGCAGAG-3′; for the reference gene - *GAPDH* (Glyceraldehyde 3-Phosphate Dehydrogenase – a housekeeping gene) forward 5′-TGGTATCGTGGAAGGACTCATGAC-3′, reverse 5′-ATGCCAGTGAGCTTCCCGTTCAGC-3′. Each reaction tube contained 5 μl RT HS-PCR Mix Sybr® B (A&A Biotechnology), 0.7 μl of 10 μM of each primer, 1 μl of cDNA template and nuclease-free water to the final volume of 10 μl. Thermal cycling conditions comprised the initial denaturation at 95 °C for 10 min and 40 cycles of denaturation at 95 °C for 10 sec, primer annealing at 56 °C for 15 sec, elongation at 72 °C for 20 sec. A negative control, without cDNA template, was included in every trial. Trials were performed in triplicate for each data point. The values obtained for the triplicates were then averaged. The *CEBPA* was normalized to the endogenous reference *GAPDH* to obtain the relative expression level of the selected gene. The 2^−ΔΔCq^ method was used to estimate relative changes in the gene expression determined by real-time PCR analysis^16^. In all subsequent data analyses delta Ct (dCt: Ct _tested gene_-Ct _reference gene_) was used instead of ratio (R: 2^−ΔΔCq^).

Statistical investigations were performed using STATISTICA 13.1 (StatSoft Inc., Tulsa, OK, USA). For verification of numerical data with normal distribution, Shapiro–Wilk test was used. To evaluate the association between the gene expression levels and selected clinical data, the T-test or r-Pearson correlation was used. In all calculations P-value ≤ 0.05 was considered statistically significant.

### Ethics approval and consent to participate

The present study was approved by The Ethics Committee of the Medical University of Lodz (number RNN/88/16/KE) and was in accordance with the principles of the Declaration of Helsinki. Written informed consent was obtained from the patients prior to their participation in the research.

## Data Availability

The datasets used and/or analysed during the current study are available from the corresponding author on reasonable request.
